# ADRD Caregiver Experiences with an Online Legal and Financial Planner

**DOI:** 10.1093/geroni/igaf122.2846

**Published:** 2025-12-31

**Authors:** Sydney Hoel, Richard Chunga, Clover Caldwell, Heather Schwartz, Matthew Zuraw, Christian Elliott, Andrew Pickett, Nicole Werner

**Affiliations:** Vanderbilt University Medical Center, Nashville, Tennessee, United States; CareVirtue, New York, New York, United States; Indiana University Bloomington, Bloomington, Indiana, United States; Indiana University Bloomington, Bloomington, Indiana, United States; Better Brains, Inc, San Diego, California, United States; CareVirtue, San Diego, California, United States; Indiana University School of Public Health- Bloomington, Bloomington, Indiana, United States; Vanderbilt University Medical Center, Nashville, Tennessee, United States

## Abstract

Legal and financial planning pose significant challenges for family caregivers of persons living with Alzheimer’s disease and related dementias (ADRD) and can lead to increased financial risk and strain. To address this, we created the CareVirtue Planner, a web-based, personalized legal and financial planning tool for ADRD caregivers. In this study, 88 ADRD caregivers completed a 3-month field test of the Planner, during which they received personalized legal and financial planning education recommendations. Participants completed semi-structured post-use interviews to share their experience of using the Planner and recommend improvements. Using an inductive team-based coding approach, we analyzed the interviews to examine participants’ experiences related to navigation, perceived usefulness of the Planner, and barriers to engagement. 70 participants were women (80%), and 64 were White (73%). Participants shared that the Planner is easy to use due to its simple and intuitive interface. Some experienced minor technical issues which impacted navigation. The Planner was most useful for early-stage caregivers; for long-term caregivers, the Planner validated their prior legal and financial planning actions but would require additional and dynamic content to facilitate continued engagement. Participants found the centralized document vault feature and personalized resource recommendations particularly useful. Suggested improvements to the Planner included additional features, e.g., the ability to share access to the document vault with others, and addressing additional topics, e.g., self-care. Future iterations of the Planner can be improved by further tailoring recommendations to participants’ stage of caregiving and expanding content and features to promote long-term use.

